# The Paratenon Contributes to Scleraxis-Expressing Cells during Patellar Tendon Healing

**DOI:** 10.1371/journal.pone.0059944

**Published:** 2013-03-26

**Authors:** Nathaniel A. Dyment, Chia-Feng Liu, Namdar Kazemi, Lindsey E. Aschbacher-Smith, Keith Kenter, Andrew P. Breidenbach, Jason T. Shearn, Christopher Wylie, David W. Rowe, David L. Butler

**Affiliations:** 1 Department of Reconstructive Sciences, College of Dental Medicine, University of Connecticut Health Center, Farmington, Connecticut, United States of America; 2 Division of Developmental Biology, Cincinnati Children's Hospital Research Foundation, Cincinnati, Ohio, United States of America; 3 Department of Orthopaedic Surgery, College of Medicine, University of Cincinnati, Cincinnati, Ohio, United States of America; 4 Biomedical Engineering Program, School of Energy, Environmental, Biological and Medical Engineering, University of Cincinnati, Cincinnati, Ohio, United States of America; National Institutes of Health, United States of America

## Abstract

The origin of cells that contribute to tendon healing, specifically extrinsic epitenon/paratenon cells vs. internal tendon fibroblasts, is still debated. The purpose of this study is to determine the location and phenotype of cells that contribute to healing of a central patellar tendon defect injury in the mouse. Normal adult patellar tendon consists of scleraxis-expressing (Scx) tendon fibroblasts situated among aligned collagen fibrils. The tendon body is surrounded by paratenon, which consists of a thin layer of cells that do not express Scx and collagen fibers oriented circumferentially around the tendon. At 3 days following injury, the paratenon thickens as cells within the paratenon proliferate and begin producing tenascin-C and fibromodulin. These cells migrate toward the defect site and express scleraxis and smooth muscle actin alpha by day 7. The thickened paratenon tissue eventually bridges the tendon defect by day 14. Similarly, cells within the periphery of the adjacent tendon struts express these markers and become disorganized. Cells within the defect region show increased expression of fibrillar collagens (Col1a1 and Col3a1) but decreased expression of tenogenic transcription factors (scleraxis and mohawk homeobox) and collagen assembly genes (fibromodulin and decorin). By contrast, early growth response 1 and 2 are upregulated in these tissues along with tenascin-C. These results suggest that paratenon cells, which normally do not express Scx, respond to injury by turning on Scx and assembling matrix to bridge the defect. Future studies are needed to determine the signaling pathways that drive these cells and whether they are capable of producing a functional tendon matrix. Understanding this process may guide tissue engineering strategies in the future by stimulating these cells to improve tendon repair.

## Introduction

Tendon injuries remain a significant socioeconomic problem that requires innovative treatment strategies for repair [1]. Compared to other tissues, the normal development and natural healing processes within tendon are poorly understood. Recently, investigators have tried to isolate tendon-specific progenitors for use in tissue-engineered repair [2], [3]. However, researchers still debate the location of cells within or around tendon that contribute to healing and whether these cells are tissue-resident progenitors [4]. This, in part, has remained a challenge because there are a limited number of known markers for the tenogenic lineage. However, recent developmental work has provided insight into molecular markers and signaling pathways that influence tendon differentiation [5]–[7]. This current study will utilize these new tools to begin to identify the cells that contribute to the tendon healing process by determining 1) their origin and 2) their expression of tenogenic markers.

As novel tenogenic markers are discovered during development, researchers have begun to analyze these markers during healing studies in the adult. For instance, the tenogenic transcription factor Scleraxis (Scx), which is expressed early in the mesenchymal lineage and remains highly expressed throughout tendon differentiation, has allowed researchers to begin to map the lineage of the cells during the healing process [8]. Following injury to the murine flexor digitorum longus tendon, Scx continues to be expressed in the native tendon tissue surrounding the wound site but not in the wound site itself, suggesting that these cells are likely not tenogenic in origin [9]. In a similar way, creating a full-length, central-third patellar tendon injury in rats causes decreased production of the matrix assembly proteins decorin and fibromodulin [10]. However, more comprehensive studies showing the spatiotemporal expression of these markers following injury are still needed.

Identifying the location and phenotype of cells that contribute to tendon healing is needed as researchers still debate whether tendon healing is driven by internal tendon fibroblasts, endotenon/epitenon fibroblasts, paratenon fibroblasts, or circulating mesenchymal cells [11]–[13]. The paratenon is thought to contribute to the healing process earlier than the epitenon or internal fibroblasts [14], [15]. This is especially true during healing of flexor tendons where the tendon sheath proliferates and forms adhesions onto the adjacent tendon surface, leading to a decrease in range of motion [15]. A better understanding of the extent by which cells from different regions of tendon contribute to the healing process and whether these cells express necessary markers for tenogenesis (ie. Scx) may benefit researchers attempting to create novel tendon repair strategies.

This study is focused on characterizing expression of numerous potentially relevant tenogenic markers during healing. These markers consist of transcription factors [scleraxis (Scx), mohawk homeobox (Mkx), early growth response 1 (Egr1) and 2 (Egr2), six homeobox 1 (Six1)], glycoproteins [tenascin-C (Tnc), tenomodulin (Tnmd)], proteoglycans [biglycan (Bgn), fibromodulin (Fmod), decorin (Dcn)], and collagens [type I collagen (Col1a1), type III collagen (Col3a1)], and smooth muscle actin alpha (SMAA). Genetic knockouts of several of these markers show impaired tendon formation [5], [16]–[18] but investigators have not fully explored the expression of these markers during healing.

Understanding both the 1) source and 2) lineage progression of cells that enter tendon wound sites following injury may provide valuable insight towards improving tendon repair strategies. Therefore, the objective of this study is to spatiotemporally measure the expression patterns of tenogenic markers following full-length, central PT injury in transgenic reporter mice up to 3 weeks post-surgery. These expression patterns during healing are characterized using GFP reporters, immunohistochemistry, and qPCR and contrasted to age-matched normal PT and contralateral shams. We hypothesize that following injury, the expression of tenogenic markers decreases in the wound site compared to normal PT and contralateral shams, leading to an impaired healing response as seen previously [19].

## Results

### Expression of Tenogenic Markers in Normal Tendon

**Figure 1:**
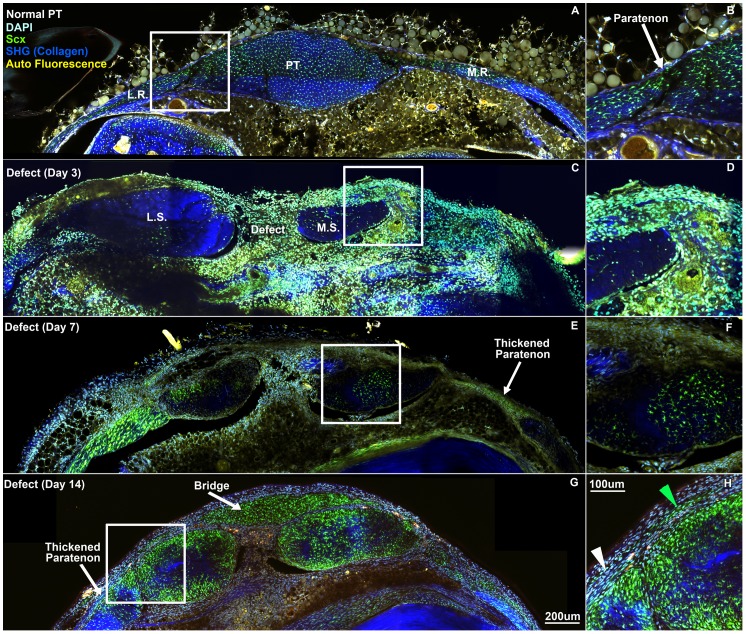
Paratenon cells produce circumferential collagen fibers as they span the defect space. The paratenon changes from a thin collagenous structure consisting of 1–2 cell layers of non-Scx-expressing cells (A–B) in normal PT to a thickened structure consisting of circumferential collagen fibers (SHG – blue; white arrowhead) and several layers of green ScxGFP cells (green arrowhead) at day 14 (G–H). As the paratenon cells migrate and bridge the defect space, the tissue is hypercellular and disorganized with a reduced SHG signal (G). PT: patellar tendon, L.R.: lateral retinaculum, M.R.: medial retinaculum; L.S.: lateral strut, M.S.: medial strut. Scale bars are 200 µm in overviews (A, C, E, G) and 100 µm in insets (B, D, F, H).

**Figure 2:**
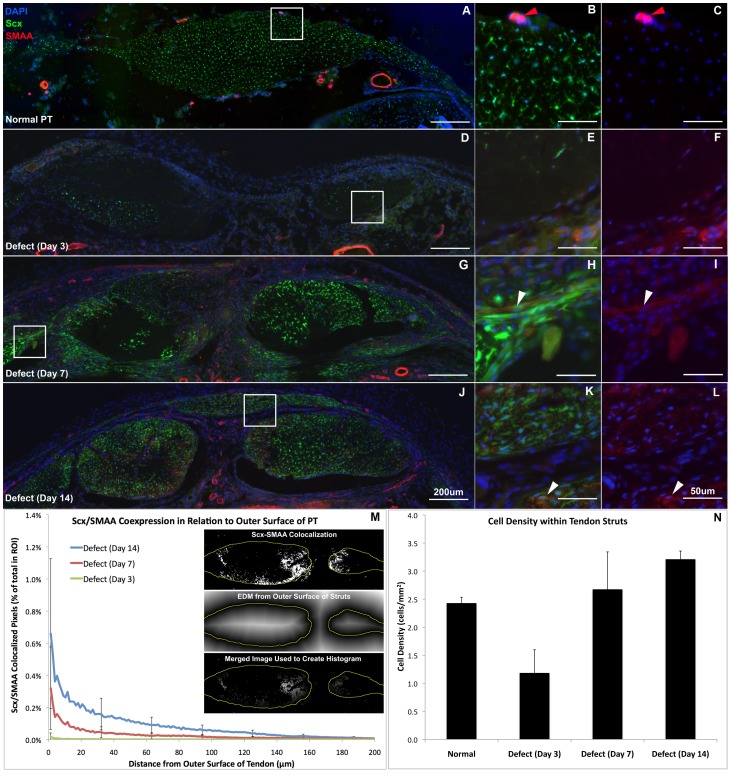
Scleraxis (Scx) and smooth muscle actin alpha (SMAA) coexpressing cells contribute to tendon healing. Cells within the thickened paratenon and adjacent struts express both Scx (green) and SMAA (red) following injury. Cells within the paratenon do not express Scx in the normal PT, but smooth muscle cells within blood vessels in the paratenon express SMAA (B–C; red arrows). Regions of high Scx and SMAA expression are within the thickened paratenon and at the anterior and posterior surfaces of the tendon struts (G–L). Scx and SMAA coexpression extends into the interior of the struts with time as seen by the EDM histograms (M). The white arrowheads point to Scx-SMAA coexpressing cells. Error bars indicate ± SD. Scale bars are 200 µm in overviews (A, D, G, J) and 50 µm in insets (B, C, E, F, H, I, K, L).

**Figure 3:**
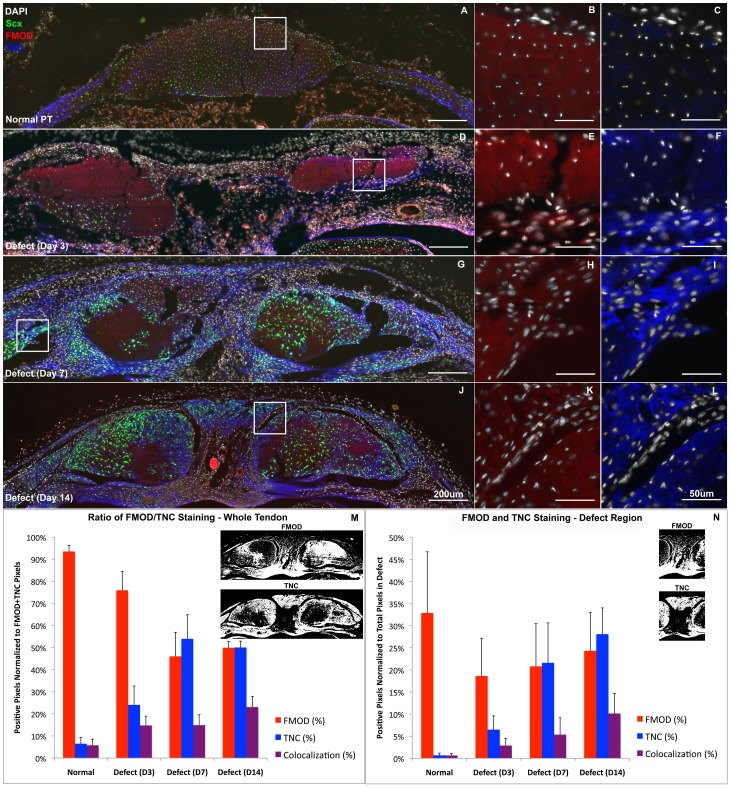
Tendon matrix transitions from predominantly fibromodulin (FMOD) to mixture of FMOD and tenascin-C (TNC). Cells within the thickened paratenon first express tenascin-C (blue) and fibromodulin (red) on day 3 (D–F) then express scleraxis (green) on days 7 and 14 (G, J). (M) Ratio of FMOD and TNC staining across the tendon width shows that the matrix transitions from close to 95% FMOD in normal tendon to a 50∶50 mixture of FMOD and TNC at day 14. (N) This relationship holds true within the defect region as well. Error bars indicate ± SD. Scale bars are 200 µm in overviews (A, D, G, J) and 50 µm in insets (B, C, E, F, H, I, K, L).

**Figure 4:**
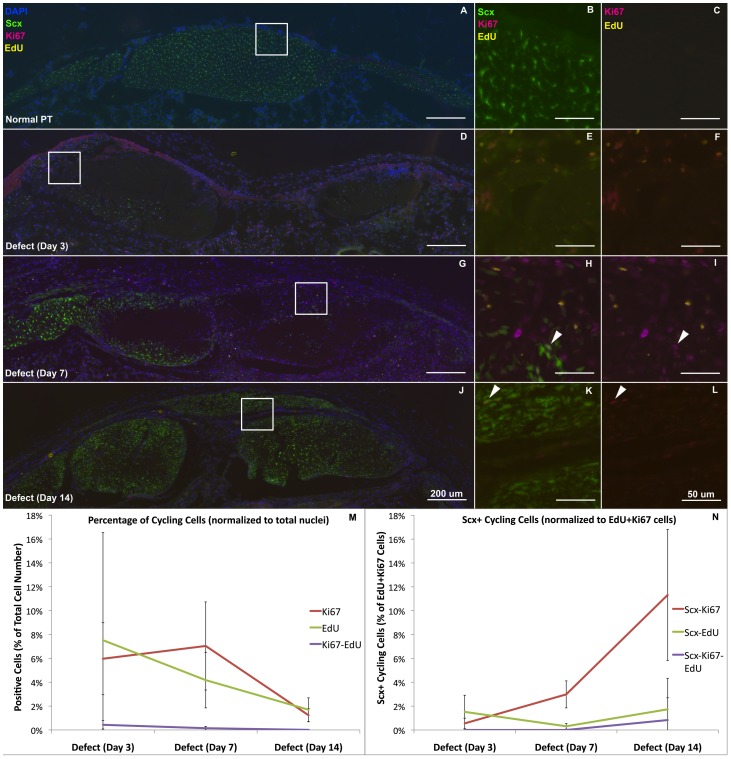
Proliferation occurs primarily in non-tenogenic cells outside of tendon. The EdU labeled cells (yellow) diminish linearly with time while the Ki67 cycling cells (red) remain consistent from days 3 to 7 then reduce at day 14 (M). However, a small subpopulation of cycling cells exists within the activated regions of paratenon and adjacent struts (white arrows), which account for less than 12% of the total cycling cells (N). The plots in the lower panels show quantification of EdU and Ki67 stained nuclei (M) and the number of cycling cells that are Scx+ (N). Error bars indicate ± SD. Scale bars are 200 µm in overviews (A, D, G, J) and 50 µm in insets (B, C, E, F, H, I, K, L).

The patellar tendon in cross section consists of ScxGFP+ cells throughout the tendon and retinaculum on the medial and lateral sides (Figs. 1–4A). Cells show a reticular morphology in this orientation due to cellular protrusions that extend out to collagen fibrils that are oriented along the tendon axis. The tendon is lined on the anterior and posterior surface with a thin paratenon that is typically only 1–2 cell layers thick with circumferential collagen fibers (Fig. 1A). The paratenon extends along the retinaculum and is continuous with the tendon. Immunostaining for SMAA shows no detectable expression within the body of the tendon and only stains smooth muscle cells within vessels in the paratenon and surrounding bursa and fat pad (Fig. 2A–C). FMOD shows consistent staining throughout the normal tendon and retinaculum with a slight decrease in staining within the interior of the tendon body (Fig. 3A–C). Minimal TNC staining is observed within the tendon body but increases along the anterior and posterior surfaces of the retinaculum (Fig. 3A). No proliferating cells (EdU or Ki67) were seen within the normal tendon, paratenon, or retinaculum (Fig. 4A–C).

### Proliferating Cells in Defect Space and Quiescent Tendon Struts at Day 3

At 3 days post-surgery, numerous EdU and Ki67 cycling cells fill the defect space (Fig. 4D). Cells within the adjacent tendon struts are not proliferating and show a similar phenotype to normal tenocytes. However, areas within the struts are devoid of cells (Fig. S1C), leading to a 50% reduction in cell density (Fig. 2N). Within the tendon and surrounding granulation tissue, 7.5% and 6% of cells stain for EdU and Ki67, respectively (Fig. 4M). However, over 88% of these stained cells do not express Scx (Fig. 4N).

### Cells within Paratenon Turn On Tenogenic Markers and Form Bridge over Defect Space

On day 3, the paratenon thickens and cells within it begin to produce FMOD and TNC (Fig. 3E–F, lower region). These cells migrate and surround the tendon struts on day 7 (Fig. 2–3G) and turn on Scx and SMAA (Fig. 2H–I, white arrowheads), while maintaining FMOD and TNC expression (Fig. 3H–I). By day 14, cells in this thickened paratenon form a bridge across the anterior surface of the defect in 75% of the samples (Figs. 1–4J). The bridge appears to be separated from the adjacent struts by a thin layer of cells not expressing Scx. The paratenon cells are oriented circumferentially over the tendon struts as opposed to cells within the tendon body which are oriented along the tendon axis. The second harmonic generation signal (SHG) (using the multi-photon microscope) shows that the paratenon cells had assembled collagen in a circumferential orientation on day 14 (Fig. 1G–H, white arrowhead). However, the lack of SHG signal in the anterior bridge at day 14 indicates that the matrix lacks collagen or is too immature to elicit a SHG signal.

### Cells within Adjacent Tendon Struts Show Delayed Response Compared to Paratenon

The cells along the periphery of the adjacent struts change from organized Scx+ cells surrounded by a matrix rich in FMOD (Fig. 3M) in normal PT to disorganized Scx/SMAA+ cells (Fig. 2–3G–L) surrounded by a matrix with an equal mixture of FMOD and TNC (Fig. 3M) at day 14. These changes originate at the tendon periphery and are delayed compared to the paratenon, with little activity seen prior to day 7. The Scx/SMAA co-expressing cells extend further into the center of the tendon struts over time (Fig. 2M).

### Contralateral Sham Tendons Show Reduced Activity

The sham injury created at the medial and lateral tendon edges displays a similar response as the defect but to a lesser extent. Cells within the paratenon and within the retinaculum near the incisions co-express Scx and SMAA (Fig. S2, white arrowheads) and are within a matrix rich in TNC (Fig. S3F,I,L). Unlike the defect groups, the paratenon response remains isolated to the retinacular incisions in the shams and doesn't extend over the surface of the PT. The cells within the tendon body of the sham group show minimal changes compared to normal PT.

### Defects Show Reduced Expression of Tenogenic Factors and Matrix Assembly Proteins

**Figure 5:**
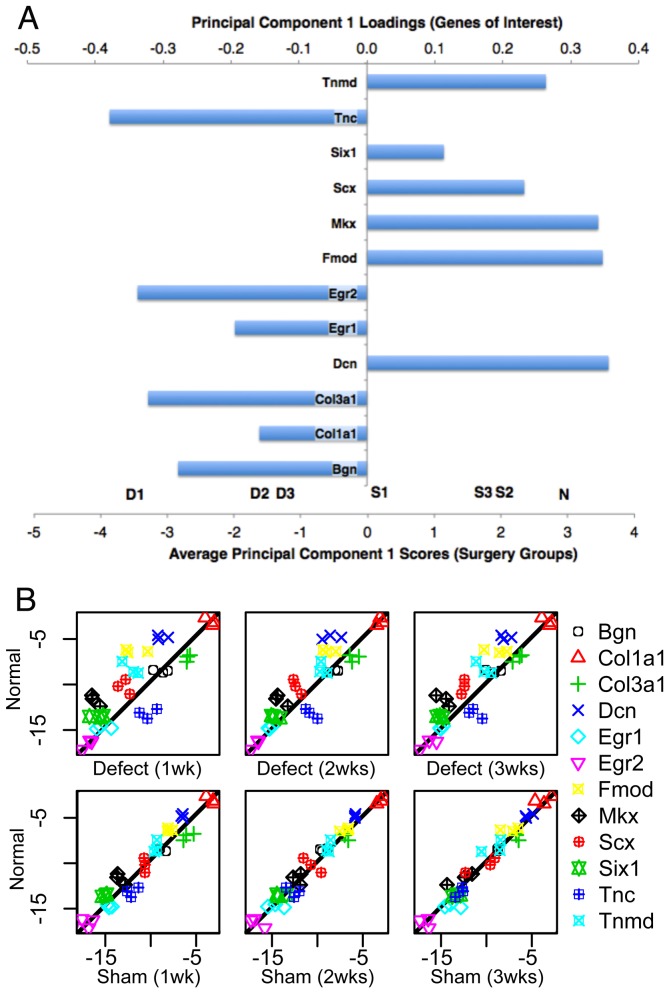
Gene expression of tenogenic transcription factors and fibril assembly proteins were reduced in the defects. Scx, Mkx, Tnmd, Dcn, and Fmod were all decreased in defects compared to contralateral shams and normal PT. (A) Principal component 1 (PC1) scores (along bottom axis) for treatment groups and loadings for genes of interest (along top axis) show genes that were up-regulated (point to left) and down-regulated (point to the right) in the defects. (B) Scatterplots depicting improved correlations with normal PT over time (horizontally) and changes in expression between the defects and shams (vertically).

Principal component analysis of the qPCR data shows that the first principal component (PC1) accounts for 45% of the variance while PC2 accounts for 32% for a total of 77% between them. PC1 effectively separates the treatment groups with the defect at 1 week having the lowest score (mean = −3.8) and normal PT having the highest score (mean = 2.9) (Fig. 5A). PC2 correlates with the mean delta C_T_ value across all genes of interest for each treatment group, rendering it an irrelevant measure of biological significance (Fig. S4). PC1 loadings for the genes of interest show that Dcn and Fmod are in the upper quartile (decreased expression in defects) while Tnc and Egr2 are in the lower quartile (increased expression in defects; Table 1; Fig. 5A).

**Table 1:** Delta C_T_ values, relative fold changes (RQ) compared to normal, and principal component 1 (PC1) loadings for each gene of interest

	Normal	Defect (1wk)		Defect (2wks)		Defect (3wks)		Sham (1wk)		Sham (2wks)		Sham (3wks)		PC1 Load
	dCt	dCt	RQ	dCt	RQ	dCt	RQ	dCt	RQ	dCt	RQ	dCt	RQ	
**Bgn**	−8.5±0.1	−6.9±0.8a	3.02	−6.7±0.6a,b	3.59	−7.9±0.8	1.52	−7.1±0.4a	2.72	−8.1±0.4b	1.32	−8.1±0.0	1.33	−0.28
**Col1a1**	−3.1±0.4	−1.8±0.5a	2.42	−1.6±0.3a	2.90	−2.2±0.6	1.83	−1.6±0.5a	2.91	−1.7±0.4a	2.71	−2.9±0.9	1.13	−0.16
**Col3a1**	−7.1±0.4	−4.2±0.2a	5.97	−4.5±0.5a,b	5.97	−5.4±0.5a	3.28	−4.2±0.6a	7.50	−5.5±0.2a,b	2.90	−5.9±0.2a	2.21	−0.32
**Dcn**	−4.8±0.2	−6.9±0.6a,b	0.24	−7.0±1.1a,b	0.23	−6.7±0.6a,b	0.28	−4.8±0.1b	1.04	−4.5±0.0b	1.24	−4.6±0.5	1.13	0.36
**Egr1**	−14.7±0.2	−13.0±0.8a	3.35	−13.8±0.2	1.94	−13.4±0.2	2.50	−12.9±0.3a	3.52	−13.5±0.9	2.33	−13.2±0.9a	2.80	−0.19
**Egr2**	−16.5±0.6	−14.5±0.5a	3.98	−15.4±0.5	2.20	−14.8±0.9	3.40	−15.3±0.6	2.27	−15.7±0.8	1.80	−16.2±0.6	1.23	−0.34
**Fmod**	−6.3±0.2	−9.8±1.2a,b	0.09	−7.3±0.8b	0.51	−7.6±1.2	0.42	−6.2±0.3b	1.08	−5.6±0.6b	1.65	−6.7±1.0	0.80	0.35
**Mkx**	−11.7±0.6	−13.8±0.5a,b	0.23	−12.6±0.7b	0.53	−13.2±0.7	0.37	−11.7±0.5b	0.98	−11.0±0.5b	1.66	−12.2±1.5	0.72	0.34
**Scx**	−10.2±0.8	−10.7±0.6b	0.70	−10.8±0.5	0.66	−11.1±0.2	0.55	−9.0±0.1b	2.36	−9.4±1.0	1.74	−9.7±1.8	1.44	0.23
**Six1**	−13.5±0.1	−13.5±0.8	1.02	−13.2±0.5	1.22	−13.6±0.3	0.97	−13.4±0.3	1.07	−13.0±0.6	1.44	−12.7±0.5	1.74	0.11
**Tnc**	−13.2±0.5	−8.3±0.9a	28.99	−9.2±0.7a,b	15.23	−9.9±0.7a,b	9.73	−10.4±0.7a	6.97	−11.5±0.8b	3.11	−12.3±0.5b	1.81	−0.38
**Tnmd**	−8.3±0.7	−10.0±0.8a,b	0.29	−7.9±0.4	1.26	−8.9±0.8	0.63	−7.7±0.2b	1.46	−7.5±0.2	1.68	−8.5±1.2	0.84	0.26

a  =  different than normal, b  =  different than defect/sham at corresponding time point (p<0.05).

MANOVA analysis indicates that although time post-surgery did not affect gene expression, surgical treatment (defect vs. sham vs. normal) did significantly alter expression at each time point post-surgery (p<0.05). Results for the genes of interest have been categorized into 3 groups: 1) fibrillar collagens, 2) transcription factors, and 3) proteoglycans and glycoproteins.

1. Fibrillar collagens. There are no differences between the defect and sham groups for either Col1a1 or Col3a1 expression. Defect and sham tissues show elevated Col1a1 and Col3a1 gene expression compared to normal tissues up to 2 weeks post-surgery (p<0.05; Table 1). However, Col1a1 expression reduces to normal levels at 3 weeks whereas Col3a1 remains elevated (p<0.05).
2. Transcription factors. The surgery groups yield variable changes in transcription factor expression (Table 1, Fig. 5A–B). Compared to shams, both Scx and Mkx are significantly reduced in the defect tissue at 1 week, with Mkx decreased at 2 weeks as well (p<0.05). Compared to unoperated normals, Mkx displays a 4-fold decrease in expression in the defect at 1 week (p<0.05) while both Scx and Mkx show reduced trends in expression at the remaining time points (p>0.05). Creating the defect also increases Egr1 and Egr2 expression compared to normal PT (p<0.05) but not when compared to sham tissues. Six1 expression did not change for either the defect or sham tissues compared to normal (p>0.05).
3. Proteoglycans and glycoproteins. Bgn and Tnc show increased expression in both the defect and sham groups. Bgn is increased at 1 and 2 weeks. By contrast, Tnc shows elevated expression at all time points, with levels approaching a 32-fold increase (p<0.05; Table 1, Fig. 5B). Fmod and Tnmd are significantly reduced compared to normal at 1 week while Dcn is reduced at all time points (p<0.05). In addition, the increase in Tnc expression and the reduction in Fmod expression compared to normal is consistent with the FMOD and TNC staining within the defect region (Fig. 3N) where FMOD staining reduces to 60–70% of normal PT while TNC increases 30–40 times on days 7 and 14. Fmod, Tnmd, and Dcn expression in the sham tissue is unchanged compared to normal PT (p>0.05).

## Discussion

The objective of this study was to determine 1) the origin and 2) phenotype of cells within the paratenon and adjacent tendon struts that contribute to the repair process following a central patellar tendon defect injury in ScxGFP mice. Previous research suggests that tendon progenitor cells may reside within the epitenon or paratenon tissue [15]. Therefore, we performed histology in transverse sections at multiple levels along the tendon length to visualize the defect, adjacent struts, and surrounding paratenon all within the same section. Viewing the healing response in this orientation shows a thickened paratenon develop far away from the injury site on the surface of the retinaculum between 3 and 7 days following the injury. The paratenon progresses from a quiescent structure of approximately 1 cell layer thick to an active, proliferative tissue of Scx, SMAA, FMOD, and TNC expressing cells that is several cell layers thick (Fig. 1G–H, Fig. 2G–L, Fig. 3G–L). The cells within the paratenon migrate toward the tendon defect on the anterior and posterior surfaces of the tendon struts, assembling circumferential collagen fibers in the process (Fig. 1G–H). The paratenon eventually forms a bridge that spans the defect space on the anterior surface of the tendon by day 14 but this matrix is still immature as it displays decreased FMOD staining and little SHG signal for collagen (Figs. 1G & 3K). These data suggest that cells within the paratenon, which normally do not express Scx, respond to injury by turning on tenogenic markers (ie. Scx), proliferating, and migrating to bridge the defect space. These cells may be a potential resident progenitor source that contributes to tendon healing but further lineage analysis is needed.

Following the thickening of the paratenon tissue, cells within the adjacent struts also express SMAA, TNC, and FMOD along with Scx. These changes originate at the periphery of the struts but progressively extend into the interior over time (Fig. 2M). Cells within these regions display reduced organization with primary alignment in the transverse plane as opposed to along the tendon axis. Although this delayed response relative to the paratenon is consistent with previous studies of tendon healing, [12], [15] only the current study presents expression of Scx and other tenogenic markers within these cells. As such, our study provides evidence that cells within the paratenon that contribute to healing express similar markers to those cells within the body of the tendon, suggesting that similar mechanisms may control the response within both the paratenon and tendon body. Future studies will investigate these mechanisms.

Creating both the injury and the surgical sham differentially affects tenogenic gene expression. The surgical defect increases Col1a1 and Col3a1 expression levels, two of the most prominent tendon fibrillar collagens. However, the surgical defect also down regulates tenogenic transcription factors known to regulate Col1a1 expression [6], [7], [20], [21] (i.e. Scx and Mkx) in the defect tissue compared to the contralateral shams, suggesting that other factors may be driving Col1a1 expression during healing. Creating the surgical defect also decreases fibromodulin and decorin, two genes important during tendon fibrillogenesis. This decreased expression may help to explain the poor mechanical integrity of the repairs seen previously [19]. These combined results suggest that while cells in healing tendons produce the same primary fibrillar collagens as normal tendon, they may lack the ability to properly assemble the synthesized proteins. Disruption in collagen assembly outside of the cell is one likely mechanism for the impaired biomechanics. Future studies will need to more closely investigate the collagen assembly process and how we can improve upon it during healing.

Other transcription factors that may modulate healing in this model include early growth response 1 and 2 (Egr1 and Egr2), which influence both development of tendon [22] and healing in several tissues including tendon [23]. Both defect and sham tissues show increased expression of Egr1 and Egr2 in this study. In addition, Egr1 increases Col1a1 gene expression following stimulation with TGFβ via Smad3 signaling in fibroblasts [24] and both of these transcription factors are implicated in promoting fibrosis [24], [25]. Egr1 also regulates Tnc expression in response to mechanical stresses [26], [27]. These genes may also be involved in wound contraction during the repair stage at 2 and 3 weeks in our model system. Further analysis is needed to determine the signaling mechanisms that regulate this response and if these correspond with decreased expression of two fibrillogenic genes (Fmod and Dcn).

Our study is not without limitations. 1) Due to limited size and frailty, it is difficult to isolate the healing tissue away from the adjacent native struts for qPCR. Therefore, contaminating tissue from the struts may have influenced gene expression results. 2) Transverse sections revealed a mixed population of cells within the defect tissue. In future studies, we plan to sort these cells using the Scx reporter to isolate the tenogenic population within the defect region. 3) EdU injections during the first 48 hours of healing did not label the Scx-expressing cells that contribute to the healing process. Instead they primarily labeled non-Scx cells within the bursa, granulation tissue, and fat pad (Fig. 4M–N). While the purpose of these injections was to label these mesenchymal cells, it suggests that proliferating cells within the circulation that migrate to the wound site during the early inflammatory stage are not mesenchymal in nature and do not assemble matrix during repair as these EdU-labeled cells are not found within the healing tissue at later time points.

We have shown that normally quiescent cells from the paratenon expand following injury and contribute to Scx-expressing cells that produce an anterior bridge over the defect space. While characterizing the lineage of cells that contribute to tendon healing is an important initial step in developing tissue engineering strategies to improve the repair process, the mechanisms that control tendon healing and how they differ from normal tenogenesis in utero still need to be elucidated. Future studies will utilize lineage-tracing experiments for relevant tenogenic markers to determine the lineage of cells that contribute to healing. Once we understand the lineage of the cells, how they contribute to the repair, and most importantly why they yield a non-functional scar, can we then expect to formulate therapeutic strategies to improve healing of these injuries.

## Methods

### Experimental Design

Tissue morphology, fluorescent reporter expression (ScxGFP), immunohistochemistry (IHC), and qPCR were investigated at 4 time points (3, 7, 14, and 21 days) post-surgery in sixty-four 20-wk old (20.5±1.6 weeks; mean±SD) mice (Table 2). Histology, immunohistochemistry, and multi-photon imaging (n = 4 each) were performed on days 3, 7, and 14 in ScxGFP mice, graciously provided to us by Dr. Ronen Schweitzer at Portland Shriners Research Center [28]. These mice were chosen as Scx is a known tenocyte marker that turns on in early tendon progenitor cells and remains expressed throughout differentiation [5], [8]. Real-time qPCR (n = 12 each) were analyzed on days 7, 14, and 21 in double transgenic pOBCol3.6GFPtpz (Col1) and pCol2-ECFP double transgenic (Col1/Col2 DT) mice, whose genetics and healing have been described previously [19], [29]. Natural healing of a full-length, central PT defect injury was directly compared with healing of contralateral shams for both histology/IHC (n = 4) and qPCR (n = 12). Inter-animal comparisons were also made for histology/IHC (n = 4 each) and qPCR (n = 12) from 16 normal 20-week old mice.

**Table 2:** Experimental Design

				Time Post-Surgery (Days)			
Treatment	Mouse Strain	Response Measure		3	7	14	21
Defect							
	ScxGFP	Histology/IHC		4	4	4	
	Col1/Col2 DT	qPCR			12*	12*	12*
Sham							
	ScxGFP	Histology/IHC		4	4	4	
	Col1/Col2 DT	qPCR			12*	12*	12*
Normal							
	ScxGFP	Histology/IHC	4				
	Col1/Col2 DT	qPCR	12*				

* Twelve animals were pooled together in 3 samples of 4 tendons each for qPCR.

### Ethics Statement

All animal procedures were approved by the Institutional Animal Care and Use Committee at the University of Cincinnati.

### Surgical Procedure

The surgical procedure was performed as previously described [19]. Briefly, the tendon was accessed through the skin and longitudinal incisions were made along the medial and lateral borders of the tendon. Jewelers forceps were then slid under the tendon and spread to tension the tendon. The lateral edge of the defect was created with a scalpel and then jewelers forceps were placed into the incision and pushed through the tendon to create the medial edge of the defect. The central strip of tissue was cut away at the proximal and distal insertions. Incisions were closed with 5–0 prolene suture (Ethicon, Cornelia, GA). For the sham procedure in the contralateral limb, longitudinal incisions along the border were made and forceps were placed under the tendon but no defect was made.

### EdU (5-ethynyl-2′-deoxyuridine) Injections

EdU is a BrdU analog that labels proliferating cells by substituting for thymidine during DNA replication in S phase. Each mouse assigned for histological analysis was injected five times with EdU (Invitrogen, Grand Island, NY). The sequence included one intraperitoneal injection at surgery (3 µg/g body weight) and two injections (morning and night) at one and two days post surgery.

### Sample Preparation for Histology and Immunohistochemistry

Following euthanasia, the femur and tibia were cut mid-shaft and the skin removed. Samples were fixed in 4% paraformaldehyde (Fisher Scientific, Pittsburgh, PA) for 24 hours at 4°C. Limbs were decalcified in 0.5M EDTA/PBS for 9–10 days at 4°C and then embedded in OCT media (Andwin Scientific, Addison, IL). Serial transverse sections (7–8 µm) were made at 5 levels spaced approximately 0.5 mm apart along the length of the tendon (total tendon length  =  3 mm) and prepared for histological analysis. Thicker (100 µm) sections were also made for multi-photon imaging. Sections were cut on a Leica CM3050S cryostat (Leica, Wetzlar, Germany) using a novel cryofilm process (Cryofilm Type 2C, Section-lab, Hiroshima, Japan) described previously [30].

### Immunohistochemistry

Frozen sections were incubated in blocking solution (5% normal goat serum, 0.1% Triton X-100, and 1% BSA in PBS) for 1 hour at room temperature. The sections were then incubated in anti-SMAA-Cy3 (1∶500; Sigma-Aldrich, St. Louis, MO), anti-fibromodulin (1∶200; AbCam, Cambridge, MA), anti-tenascin-C (1∶1000; AbCam), or anti-ki67 (1∶200; AbCam) primary antibodies either 1 hour at room temperature or overnight at 4°C. Sections were then washed 3X in PBS before being incubated in secondary antibodies (Alexa Fluor IgG; Invitrogen). Slides were washed again, counterstained with DAPI nuclear stain, mounted, and photographed with the Zeiss Imager Z1 microscope (Carl Zeiss, Thornwood, NY). After imaging, coverslips were lifted off the slides in PBS, stained for EdU following the manufacturer's protocol (C10269; Invitrogen), remounted, and imaged again.

### Multi-Photon Imaging

Thick (100 µm) transverse sections were incubated with 0.1% Triton X-100 in PBS for 1 hour at room temperature and then stained with DAPI in 0.1% Triton X-100 at room temperature for 1 hour. Each section was then mounted with 50% glycerol and imaged on the multi-photon microscope (Ultima IV, Prairie Technologies, Middleton, WI). Samples were scanned at 1 µm increments through the full thickness of the section at an excitation wavelength of 900 nm. Data was collected at 4 channels with the following bandpass filters: 435–485 nm, 500–550 nm, 570–620 nm, and 640–680 nm.

### Image Quantification

#### Scx-SMAA Colocalization Distance Mapping (Fig. 2M)

Populations of Scx-SMAA coexpressing cells appear following injury. Euclidean distance mapping (EDM) was used within Fiji (image analysis software based on ImageJ; version 1.47) [31] to quantify the distance these cells were away from the tendon periphery. Grayscale (8-bit) images for ScxGFP and SMAA immunostaining were thresholded to equivalent values across treatment groups. These thresholded images were then overlaid to create a colocalized binary image. Distant maps were created from lines drawn along the periphery of the tendon struts (Fig. 1), which created a gradient from 0 (drawn line on tendon periphery) to 255 (255 pixels away from tendon periphery). The distance map and colocalized image were then overlaid in order to quantify the distance away from the tendon periphery for each colocalized pixel. A histogram was formed from this image and the pixels were converted to distance (µm) for quantitative analysis.

#### Cellular Density within Tendon Struts (Fig. 2N)

The lines drawn along the tendon periphery (see Fig. 2M) were used to create a mask to segment the tendon body away from the surrounding tissue. This mask was applied to grayscale (8-bit) DAPI images. The resultant images were thresholded to equivalent values and the nuclei were further segmented via the watershed function in Fiji. The nuclei were counted with the automated particle analyzer in Fiji and were normalized to unit area (mm^2^).

#### FMOD-TNC Colocalization (Fig. 3M-N)

The FMOD and TNC staining was quantified within the defect region and surrounding tissues during healing. The staining was quantified within two regions of interest (ROI): 1) a rectangular region that included the entire tendon width and 2) the defect region, which was based on average defect width (0.63 mm). Grayscale (8-bit) images for FMOD and TNC staining were thresholded to equivalent values across treatment groups. These images were also overlaid to create colocalized images. The number of pixels were then quantified and normalized to the total number of FMOD and TNC positive pixels (ROI 1) or to the total number of pixels (ROI 2).

#### Proliferating Cells (Fig. 4M-N)

The number of EdU and Ki67 positive cells were quantified in order to chase EdU labeled cells up to 14 days following injury and also to determine if these cells were still cycling (Ki67). Both of these stains are nuclear, therefore grayscale images for EdU, Ki67, and DAPI were all thresholded at equivalent levels across all treatment groups to identify positive cells. The DAPI thresholded image was overlaid on the EdU and Ki67 images to create binary images for EdU and Ki67 positive nuclei, respectively. EdU and Ki67 colocalized nuclei images were also created. These images were also overlaid on the Scx channel to count the number of proliferating Scx-expressing cells. These positive nuclei were then counted using the automated particle counter within Fiji.

### Quantitative Real-Time PCR (qPCR)

Following euthanasia, the defect, sham, and normal tendon tissues were isolated and stored in RNAlater*®* (Invitrogen) solution at −20°C. RNA was then isolated from the samples using the RNAqueous*®*-4PCR kit (AM1914; Invitrogen) following manufacturer's protocol. Briefly, samples were transferred to a fresh tube with lysis/binding solution and then disrupted with a pestle and vortexed vigorously. The lysate was then captured in the glass filter, washed, and eluted. RNA was quantified using the Qubit*®* RNA assay kit (Invitrogen) and converted to cDNA using the High Capacity RNA-to-cDNA kit (4387406; Applied Biosystems, Grand Island, NY). Real-time reactions were performed using Taqman*®* Gene Expression Mastermix (4369510; Applied Biosystems) and Taqman*®* probes for Scx, Mkx, Egr1, Egr2, Six1, Tnmd, TnC, Fmod, Dcn, Bgn, Lum, Col1a1, and Col3a1 (Table S1). The relative amount of mRNA for each gene of interest was computed using ΔC_T_ values normalized to the average of 18S and Gapdh.

### Statistical Analysis

Delta C_T_ values for the normal, defect and sham groups at 1, 2, and 3 weeks were analyzed using principal component analysis (PCA) using the R statistical package. PCA is an ordination method that reduces the dimensionality of multivariate data into principal components by producing linear combinations of the original data variables in order to summarize the variance of the data [32]. In addition, PCA allows the researcher to determine variables that correlate with each other. Delta C_T_ values were also compared for each gene of interest via two-way MANOVA analysis with surgical treatment and time post-surgery as fixed factors (p<0.05; Table 1). Image quantification values were analyzed via ANOVA with time as fixed factor (p<0.05). ANOVAs were conducted using SPSS 13.0 (Chicago, IL).

## Supporting Information

Figure S1 Following the defect injury, hematoxylin staining shows that granulation tissue fills the defect space by day 3 (C) and regions of the adjacent struts were devoid of cells. At later time points, the struts repopulated with cells starting at the tendon surface (E) and extending further into the interior by day 14 (G). Cells within the paratenon produced a bridge that spanned the defect space by day 14. Scale bars are 200 µm in overviews (A, C, E, G) and 50 µm in insets (B, D, F, H).
(TIF)

Figure S2 Tendon midsubstance of contralateral shams showed little activity while Scx and SMAA positive cells in the paratenon worked to repair the injuries to the retinaculum. Scale bars are 200 µm in overviews (A, D, G, J) and 50 µm in insets (B, C, E, F, H, I, K, L).
(TIF)

Figure S3 Tendon midsubstance of contralateral shams showed consistent FMOD staining with little TNC, which was comparable to normal PT. However, paratenon cells expressed both FMOD and TNC in response to the injury at the retinaculum on the tendon borders. Scale bars are 200 µm in overviews (A, D, G, J) and 50 µm in insets (B, C, E, F, H, I, K, L).
(TIF)

Figure S4 Scatterplot of the mean Delta C_T_ value across all twelve genes of interest (GOIs) vs the principal component (PC2) scores for each treatment group. These values were highly correlated (R = −0.99). This shows that PC2, while contributing to 32% of the total variance, is not of biological significance since it models for the mean value across all 12 GOIs.
(TIF)

Table S1 Taqman probe assay ID for each gene of interest.
(DOC)
