# Supplementary figures and images for: The Paratenon Contributes to Scleraxis-Expressing Cells during Patellar Tendon Healing
Source: PLoS One. 2013 Mar 26;8(3):e59944. doi: 10.1371/journal.pone.0059944 (PMC3608582; doi:10.1371/journal.pone.0059944)

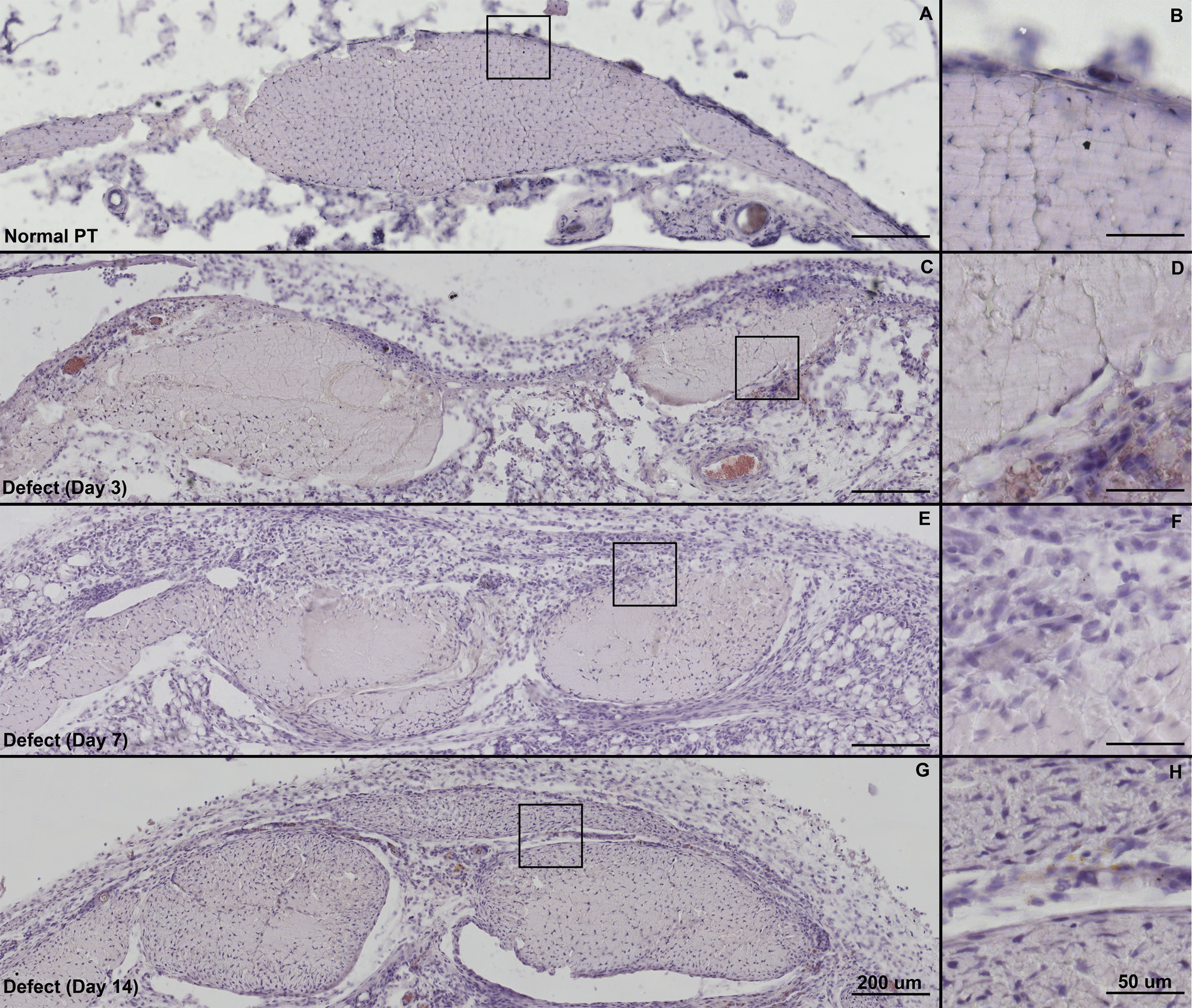

Supplement: Figure S1 — Following the defect injury, hematoxylin staining shows that granulation tissue fills the defect space by day 3 (C) and regions of the adjacent struts were devoid of cells. At later time points, the struts repopulated with cells starting at the tendon surface (E) and extending further into the interior by day 14 (G). Cells within the paratenon produced a bridge that spanned the defect space by day 14. Scale bars are 200 µm in overviews (A, C, E, G) and 50 µm in insets (B, D, F, H). (TIF) [file pone.0059944.s001.tif]

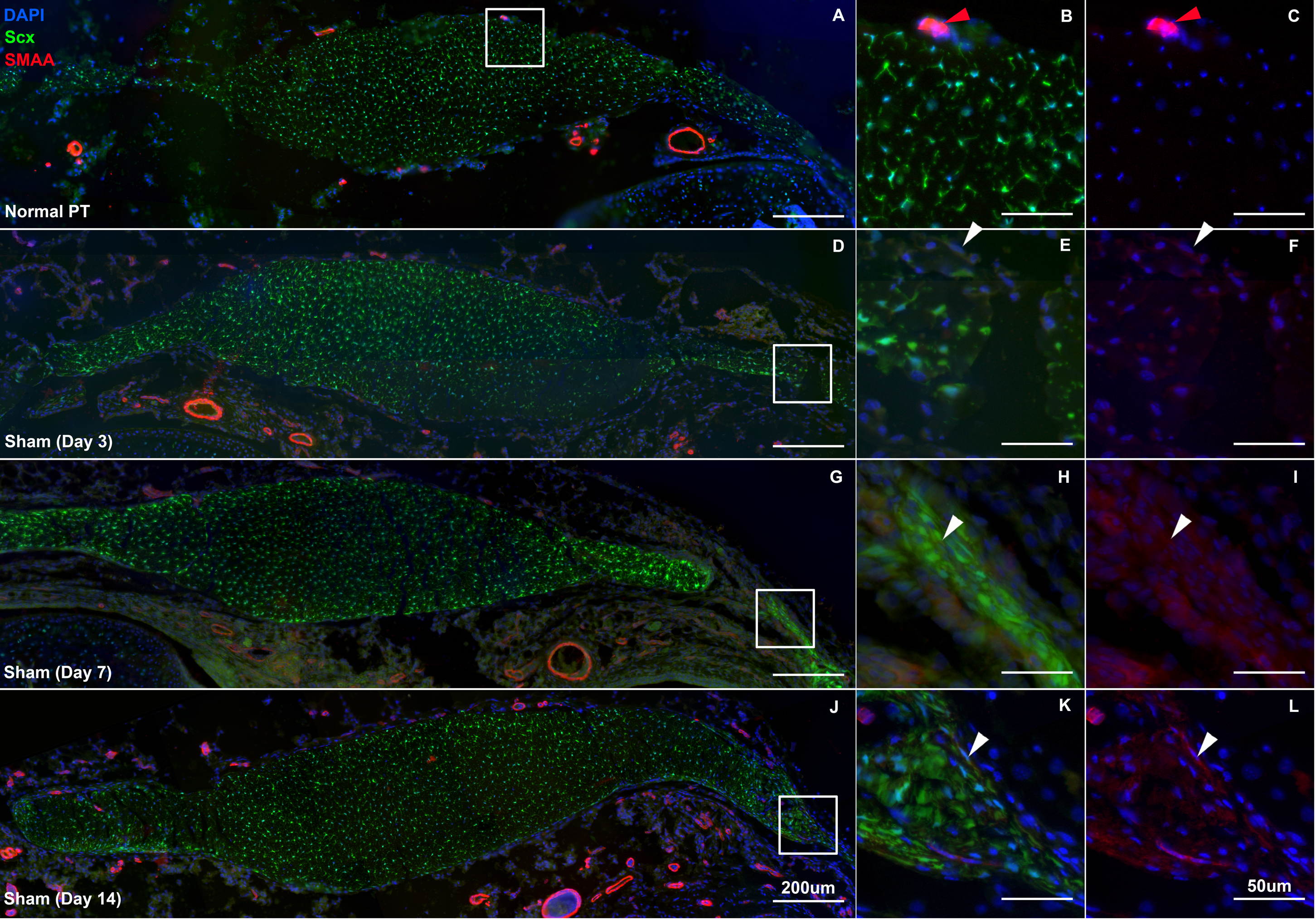

Supplement: Figure S2 — Tendon midsubstance of contralateral shams showed little activity while Scx and SMAA positive cells in the paratenon worked to repair the injuries to the retinaculum. Scale bars are 200 µm in overviews (A, D, G, J) and 50 µm in insets (B, C, E, F, H, I, K, L). (TIF) [file pone.0059944.s002.tif]

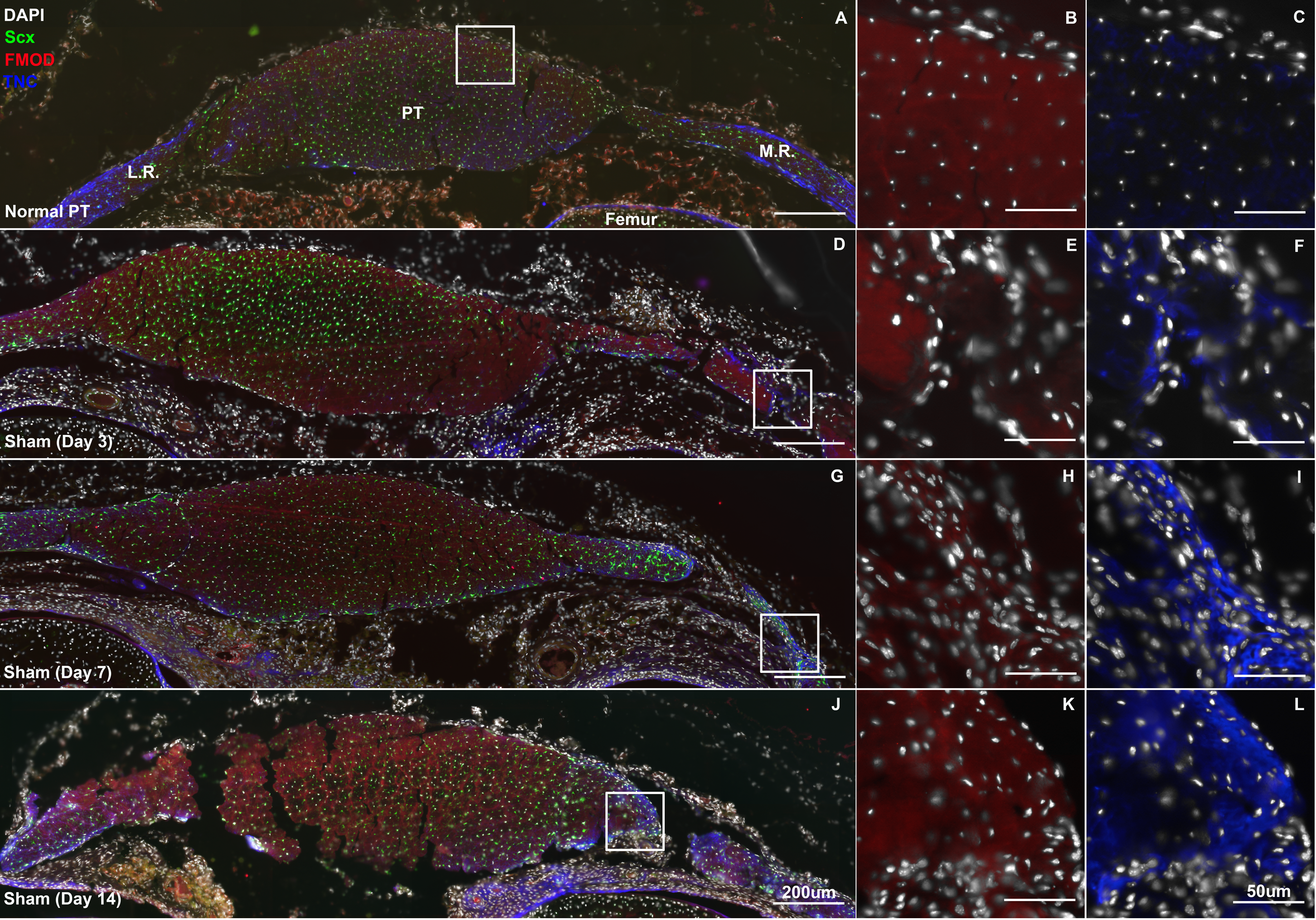

Supplement: Figure S3 — Tendon midsubstance of contralateral shams showed consistent FMOD staining with little TNC, which was comparable to normal PT. However, paratenon cells expressed both FMOD and TNC in response to the injury at the retinaculum on the tendon borders. Scale bars are 200 µm in overviews (A, D, G, J) and 50 µm in insets (B, C, E, F, H, I, K, L). (TIF) [file pone.0059944.s003.tif]

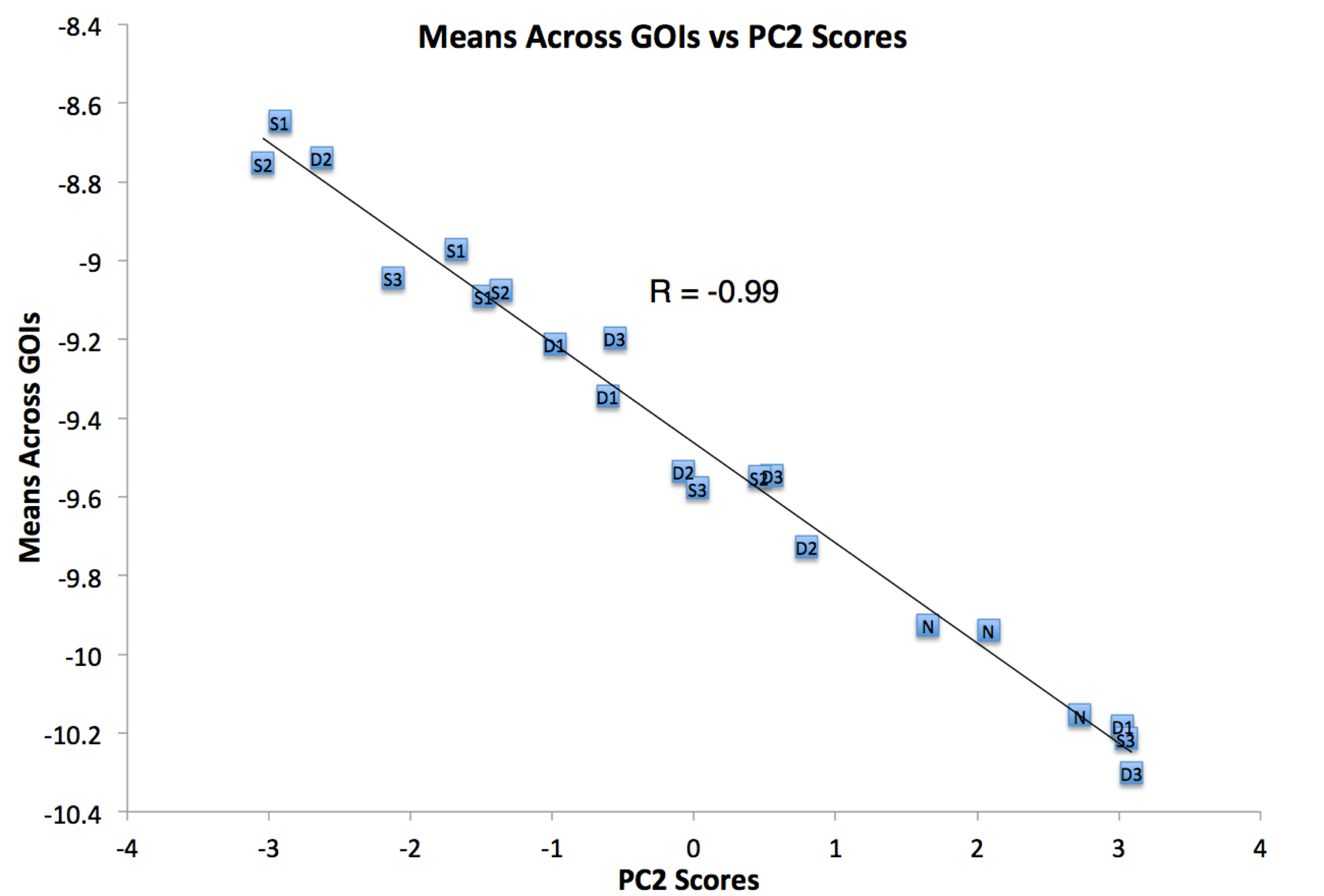

Supplement: Figure S4 — Scatterplot of the mean Delta CT value across all twelve genes of interest (GOIs) vs the principal component (PC2) scores for each treatment group. These values were highly correlated (R = −0.99). This shows that PC2, while contributing to 32% of the total variance, is not of biological significance since it models for the mean value across all 12 GOIs. (TIF) [file pone.0059944.s004.tif]
