# Supplementary material for: The Paratenon Contributes to Scleraxis-Expressing Cells during Patellar Tendon Healing
Source: PLoS One. 2013 Mar 26;8(3):e59944. doi: 10.1371/journal.pone.0059944 (PMC3608582; doi:10.1371/journal.pone.0059944)
Supplement: Table S1 — Taqman probe assay ID for each gene of interest. (DOC) [file pone.0059944.s005.doc]

**Table S1.** Taqman probe assay ID for each gene of interest.

| **Gene Name** | **Gene Symbol** | **Assay ID** |
| --- | --- | --- |
| Eukaryotic 18S rRNA | 18S | Hs99999901_s1 |
| Glyceraldehyde-3-phosphate dehydrogenase | Gapdh | Mm99999915_g1 |
| Scleraxis | Scx | Mm01205675_m1 |
| Mohawk homeobox | Mkx | Mm00617017_m1 |
| Early growth response 1 | Egr1 | Mm00656724_m1 |
| Early growth response 2 | Egr2 | Mm00456650_m1 |
| Sine oculis-related homeobox 1 homolog | Six1 | Mm00808212_m1 |
| Biglycan | Bgn | Mm00455918_m1 |
| Fibromodulin | Fmod | Mm00491215_m1 |
| Tenomodulin | Tnmd | Mm00491594_m1 |
| Decorin | Dcn | Mm00514535_m1 |
| Tenascin-C | Tnc | Mm00495662_m1 |
| Type I collagen, alpha 1 chain | Col1a1 | Mm00801666_g1 |
| Type III collagen, alpha 1 chain | Col3a1 | Mm01254476_m1 |
